# Inhibition of NF-κB Pathway and Modulation of MAPK Signaling Pathways in Glioblastoma and Implications for Lovastatin and Tumor Necrosis Factor-Related Apoptosis Inducing Ligand (TRAIL) Combination Therapy

**DOI:** 10.1371/journal.pone.0171157

**Published:** 2017-01-30

**Authors:** Pi Chu Liu, Gang Lu, Yi Deng, Cheng Dong Wang, Xian Wei Su, Jing Ye Zhou, Tat Ming Chan, Xiang Hu, Wai Sang Poon

**Affiliations:** 1 Division of Neurosurgery, Department of Surgery, The Chinese University of Hong Kong, Hong Kong, China; 2 Shenzhen Key Laboratory of Cell Microenvironment, Department of Biology, South University of Science and Technology of China, Shenzhen, Guangdong, China; 3 Shenzhen Beike Cell Engineering Research Institute, Shenzhen, China; University of Navarra, SPAIN

## Abstract

Glioblastoma is a common malignant brain tumor and it is refractory to therapy because it usually contains a mixture of cell types. The tumor necrosis factor-related apoptosis inducing ligand (TRAIL) has been shown to induce apoptosis in a range of tumor cell types. Previously, we found that two human glioblastoma cell lines are resistant to TRAIL, while lovastatin sensitizes these glioblastoma cells to TRAIL-induced cell death. In this study, we investigated the mechanisms underlying the TRAIL-induced apoptosis in human glioblastoma cell lines by lovastatin. Furthermore, we have confirmed the anti-tumor effect of combination therapy with lovastatin and TRAIL in the subcutaneous brain tumor model. We showed that lovastatin significantly up-regulated the expression of death receptor 5 (DR5) in glioblastoma cell lines as well as in tumor-bearing mice with peri-tumoral administration of lovastatin. Further study in glioblastoma cell lines suggested that lovastatin treatment could inhibit NF-κB and Erk/MAPK pathways but activates JNK pathway. These results suggest that lovastatin sensitizes TRAIL-induced apoptosis by up-regulation of DR5 level via NF-κB inactivation, but also directly induces apoptosis by dysregulation of MAPK pathway. Our *in vivo* study showed that local peri-tumoral co-injection of lovastatin and TRAIL substantially reduced tumor growth compared with single injection of lovastatin or TRAIL in subcutaneous nude mice model. This study suggests that combined treatment of lovastatin and TRAIL is a promising therapeutic strategy to TRAIL-resistant glioblastoma.

## Introduction

Cancer is a class of diseases characterized by abnormal cell proliferation and survival, which are closely associated with dysregulated programmed cell death or apoptosis[1]. Apoptosis has gained considerable interest as a promising therapeutic target in cancer therapy. Signaling pathways that control the apoptotic process are therefore amenable to pharmacological intervention for tumor progression. One of the pathways that trigger the initiation of apoptosis is mediated through death receptors (DR) on the cell surface. Eight death receptors have been characterized so far, including TNF-related apoptosis-inducing ligand (TRAIL) receptor 1 (TRAILR1/DR4) and TRAILR2/DR5[2, 3]. The binding of natural death ligands (TNF cytokines) to DR4 or DR5 triggers the formation of death-inducing signaling complex (DISC)[4], which involves oligomerization of the DR and recruitment of Fas-associated death domain protein (FADD), proapoptotic caspase 8–10 as well as antiapoptotic cellular FADD-like IL-1β-converting enzyme-inhibitory protein (cFLIP), via homotypic protein-protein interactions between their death domains. The integration of the pro- and anti-apoptosis signals eventually leads to life-or-death decision making. In addition, decoy receptors (DcRs) that lack functional death domains also interact with death ligands, but do not trigger the formation of signaling complexes[3].

The discovery and early *in vitro* studies of TRAIL signaling pathway have shed light on the cancer treatment; however, subsequent clinical studies revealed weak therapeutic effects[5]. Many human cancer types such as glioblastoma are resistant to TRAIL-targeted therapies[5]. Glioblastoma is the most common and highly malignant brain cancer. Given that glioblastoma usually contains a mix of cell types with varied susceptibility to certain therapies, it is highly refractory to treatment^6^. Therefore, several combined treatment regimens could be used for therapeutics in glioblastoma patients[6].

Recently, we reported that lovastatin, a widely used cholesterol-lowering agent for prevention of atherosclerotic cardiovascular diseases, sensitized human glioblastoma cells to TRAIL-induced apoptosis and caused cell cycle arrest at G0/G1 phase[7]. However, the underlying mechanisms remain elusive. Here we demonstrated that lovastatin treatment elevates DR5 expression in all four glioblastoma cell lines including grade IV glioblastoma multiforme (GBM) cell line U87 derived from high-grade gliomas, which are intrinsically TRAIL-resistant. *In vitro* experiments indicated that this was likely mediated by the inhibition of NF-κB and/or activation of stress-activated protein kinases pathways. Using subcutaneous brain tumor mouse models, we consistently showed that *in vivo* lovastatin treatment also induced DR5 expression in the tumor tissue and inhibited tumor growth; importantly combined treatment with lovastatin and TRAIL resulted in synergistic effects that does not only inhibit tumor growth, reduce tumor volume, but also inhibit Erk activation in U87 cell line. Our results provide molecular basis and pre-clinical evidence that lovastatin potentiates efficacy of TRAIL-based therapy for the treatment of human glioblastoma.

## Materials and Methods

### Ethics statement

The primary GBM tissues used in this study were resected from patients with GBM who were recruited at the Prince of Wales Hospital, an affiliated teaching hospital of The Chinese University of Hong Kong. This study was approved by The Joint Chinese University of Hong Kong–New Territories East Cluster Clinical Research Ethic Committee, and written informed consent were obtained.

### Cell lines and cell culture

Four glioblastoma cell lines A172, M059J, M059K, and U87, and HEK293T cells were purchased from the American Tissue Culture Collection (USA). A172, M059J, and M059K cell lines were maintained as described previously[7]. U87 cells were grown in Eagle’s minimum essential medium (MEM) (Gibco, USA), while HEK293T cells were grown in Dulbecco’s modified Eagle’s medium (DMEM) (Gibco, USA). All culture mediums were supplemented with 10% FBS (HyClone, USA), 100 U/mL penicillin (Gibco, USA), and 100 g/mL streptomycin (Gibco, USA) and cells were cultured at 37°C with 5% CO_2_.

### MTT assay

5×10^3^ cells were seeded into 96-well plate with appropriate complete medium. After incubation with lovastatin and TRAIL, the medium was removed and replenished by 100 μl of working MTT solution (Promega). The MTT working solution was 1:10 diluted of stock (5 mg/ml, Promega) in medium. The cells were then incubated at 37°C in 5% CO_2_ for 3 hours. Then the MTT solution was replaced by 100 μl of DMSO. The plate was shaken gently and the absorbance was measured at 570 nm with a reference of 630 nm using a microplate reader (Spectra Rainbow, TECAN). Cell viability was determined as the percentage of Absorbance of the treatment vs. Absorbance of the control.

### Annexin V and propidium iodide (PI) staining, and cell cycle analysis

2×10^**5**^ cells were seeded into 60-mm plates with appropriated complete medium. Annexin V fluorescence dye (Molecular Probe, USA) and PI (Molecular Probe, USA) staining, and cell cycle analysis were carried out as described previously[7].

### Western blotting

Western blot analysis was performed as previously described[7]. Total protein was extracted from the cultured cells using a cell scraper and RIPA buffer [1×PBS, 1% Nonidet P-40, 0.5% sodium deoxycholate and 0.1% sodium dodecyl sulfate, pH 7.6 with 1×Protease inhibitors (Roche) and 100 μg/ml of PMSF]. The protein samples were separated by SDS-PAGE and then transferred to nitrocellulose blotting membranes. The membranes were blocked in 5% non-fat milk for 1 hour, probed with primary antibodies overnight at 4°C, and then incubated with secondary antibody for 1 hour at room temperature. The membrane was then infiltrated with chemiluminescence to detect the target protein signal.

Primary antibodies against DR5, DcR1, NF-κB p65, phosphor-NF-κB p65, IκBα, phosphor-IκBα, phosphor-IKKα/β, JNK, phosphor-JNK, phosphor-p38, Erk, or phosphor-Erk were purchased from Cell Signaling (USA). Antibodies against p38 and horseradish peroxidase conjugated anti-mouse-IgG or anti-rabbit-IgG were from Santa Cruz (USA). The primary antibodies were diluted to 1:1000 and the secondary antibodies were diluted to 1:2000.

### Animals and drug treatment

All animal procedures were performed in accordance with the guidelines for care and use of laboratory animals and approved by The Animal Subjects Committee of The Chinese University of Hong Kong. Male 4-week-old BALB/c nude mice (n = 30) were obtained from Laboratory Animal Services Center, The Chinese University of Hong Kong and the animals were housed in specific pathogen-free conditions with a 12 hour light-dark cycle and free access to water and food. 5×10^6^ U87-GFP-Luc cells were injected subcutaneously into the dorsal region of the mice. The body weight, tumor size, and other general physiological conditions were assessed every 2 days. The tumor volume (V) was determined as follows: V = 0.5 × a × b^2^, where a is the length and b the width of the tumor. For euthanasia of mouse, overdose of sodium pentobarbital was used by intraperitoneal injection (dosage >100 mg/kg body weight).

Lovastatin was from Toronto Research Chemicals (Canada). It was dissolved in DMSO for *in vitro* experiments. For *in vivo* treatment, 40 mg of lovastatin was dissolved in 12.5 mL of 96% ethanol and then mixed with 18 mL of 0.1 M NaOH. The mixture was incubated at 50°C for 2 hrs. After incubation, the dissolved lovastatin was adjusted to pH 7.0 with 0.1 M HCl. The lovastatin stock solution was diluted in distilled water for serial dilutions. TRAIL was purchased from PeproTech (USA). It was dissolved in 1× PBS at a concentration of 2 μg/μl.

For drug treatments, lovastatin (5 mg/kg or 10 mg/kg) was given daily by intraperitoneal injection or peri-tumoral injection for 12 days. The mice receiving co-treatment of lovastatin and TRAIL were treated with lovastatin daily and peri-tumoral administrated with TRAIL (200 ng) from day 4 to 6, and day 10, and day 12 post treatment. During the treatment, the body weight, tumor size, and other general conditions were assessed every 2 days. IVIS images were taken twice a week.

### In vivo imaging system (IVIS) imaging

Mice were anesthetized with isoflurane (Alfamedic, Hong Kong) at least 7 minutes before luciferin injection. Luciferin (150 mg/kg) was delivered by intraperitoneal injection, and images of tumor-bearing nude mice were taken by in vivo imaging system (Caliper LifeScience, USA).

### Immunohistochemistry

Tumor specimens were fixed in 10% paraformaldehyde and processed for tissue sectioning. Paraffin-embedded sections (5 μm) were then de-waxed and hydrated. 3% H_2_O_2_ was used to block the endogenous peroxidase. After blocking in 10% normal blocking serum, the slides were incubated with Ki-67 (Santa Cruz, 1:50) or DR5 (CST, 1:50) antibody overnight at 4°C. The slides were then washed and incubated with specific biotinylated secondary antibody (Vector, 1:100) for 1.5 hours at room temperature. After washing, the slides were then incubated with horseradish peroxidase-conjugated streptavidin (Vector, 1:200) for 1.5 hours at room temperature. Finally, the tissues were stained using DAB-plus reagent kit (Thermo Scientific).

### Statistical analysis

Data were presented as means ± SD (n ≥ 3). Differences between groups were examined for statistical significance using Student’s t test or one-way ANOVA. *p* < 0.05 was considered to be statistically significant.

The method of generating U87-GFP-Luc cell lines and dual-luciferase reporter assay can be found within Supplementary Materials and Methods.

## Results

### Lovastatin sensitizes glioblastoma cell lines to TRAIL-induced apoptosis and promotes DR5 expression

Numerous cancers have previously been shown to be refractory to TRAIL treatment[8–11]. We have recently reported that three human malignant glioblastoma cell lines (M059J, M059K, and A172) are resistant to TRAIL-induced apoptosis; interestingly, treatment of lovastatin in combination with TRAIL significantly induced apoptotic cell death[7].

To further examine the synergistic effect of lovastatin and TRAIL, we tested the grade IV GBM cell line U87 derived from high-grade gliomas and evaluated the effects of lovastatin on TRAIL-induced apoptosis. Similar to M059J, M059K, and A172 cell lines, U87 cells consistently exhibited strong resistance against TRAIL as revealed by MTT assay (S1A Fig). TRAIL treatment at either low (100 ng/mL) or high (200 ng/mL) dose for 24, 48 or 72 hours did not promote cell death of U87 (S1A Fig); in contrast, the combined treatment with TRAIL (100 ng/mL) and lovastatin significantly decreased cell viability in a dose-dependent manner (S1B Fig). Lovastatin alone, however, did not significantly affect cell viability under the same conditions (S1B Fig). Co-treatment with TRAIL (100 ng/mL) and lovastatin (20 μM) also caused cell shrinkage, morphological changes and cell aggregation (S1C Fig). We then measured apoptotic cell death by Annexin V assay. In contrast to cells treated with TRAIL or lovastatin alone, synergistic induction of apoptotic cell death was detected when cells were treated with TRAIL (100 ng/mL) and lovastatin (20 μM) over 48-hour treatment (S1D Fig). Taken together, lovastatin can effectively and broadly sensitize GBM cell lines to TRAIL-induced apoptosis.

**Fig 1:**
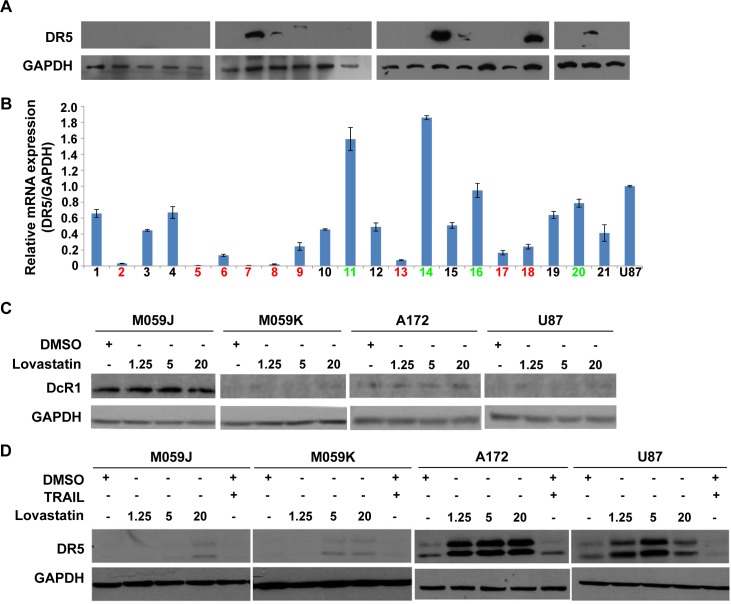
The TRAIL receptor DR5 is up-regulated in glioblastoma cell lines after lovastatin treatment. (A) We collected GBM primary brain tumor samples from 21 patients and measured the DR5 expression by western blot. 7 out of 21 GBM primary samples exhibit strong or detectable expression of DR5. (B) The mRNA level of DR5 transcripts in the 21 GBM primary samples was also examined by quantitative PCR (normalized to GAPDH). The DR5 expression in U87 cell line was used as reference. The samples with high expression of DR5 were marked by green, and the samples with weak DR5 expression were marked by red. (C-D) Four human glioblastoma cell lines were treated with various concentrations of lovastatin, and then the expression of DcR1 (C) and DR5 (D) was detected with western blot. Representative western blots were shown.

Given that TRAIL-triggered apoptosis is mediated through death receptors (DR) on the cell surface. We examined the expression of DR5 in human GBM primary samples. Only 7 out of the 21 samples exhibited strong or detectable DR5 expression (Fig 1A). In mRNA level, only 4 out of the 21 GBM primary samples showed similar or higher level of DR5 expression compared to U87 cell line (Fig 1B, samples 11, 14 and 16). In 9 samples, the expression level of DR5 was low or nearly undetected (Fig 1B). For the remaining 8 samples, DR5 expression level was around half of that in U87 cell line (Fig 1B). Weak or undetectable DR5 expression could partly explain the TRAIL resistance in GBM. In addition, it was postulated that the competitive binding of decoy receptors DcR1 and DcR2 to TRAIL confers cancer cells resistance to TRAIL-induced apoptosis. We next measured the expression of decoy receptors and death receptors in the four TRAIL-resistant cell lines including M059J, M059K, A172, and U87 cells that were treated with different concentrations of lovastatin. Lovastatin treatment did not alter the expression level of decoy receptors in these cell lines (Fig 1C; data for DcR2 not shown). In contrast, lovastatin statistically up-regulates DR5 expression dose-dependently in all four cell lines, particularly in A172 and U87 cell lines (Fig 1D). These findings indicated that lovastatin treatment promotes DR5 expression in multiple human glioblastoma cell lines, which provides a molecular basis by which lovastatin sensitizes TRAIL-induced apoptosis in GBM.

### Lovastatin inhibits NF-κB pathway in glioblastoma cell lines

**Fig 2:**
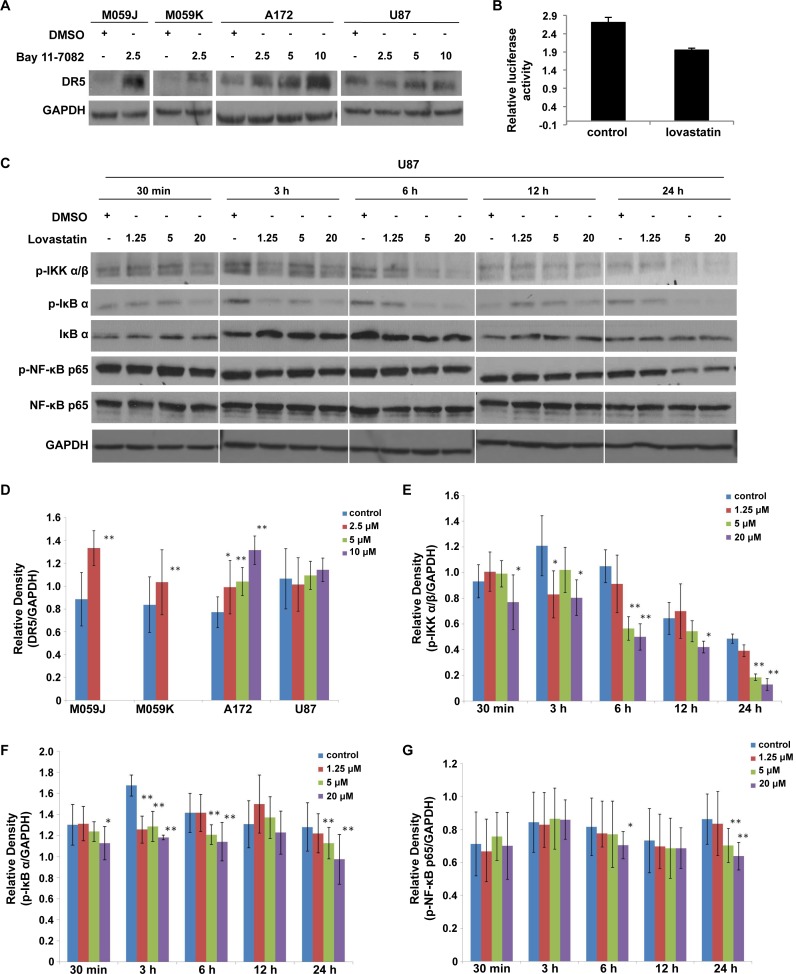
Lovastatin treatment in U87 cell line attenuates NF-κB pathway. (A, D) The four glioblastoma cell lines were treated with Bay 11–7082. After treatment with Bay for 48 hours, the expression of DR5 was up-regulated in the glioblastoma cell lines. The relative densities of DR5 bands normalized to GAPDH were shown (D). (B) Dual-luciferase reporter assay showed that incubation with 20 μM lovastatin for 6 hours attenuated the activity of NF-κB pathway in U87 MG cells. (C, E-G) U87 MG cells were treated with different concentrations of lovastatin for 30 minutes, 3, 6, 12 or 24 hours. Western blot was used to detect the expression of NF-κB p65, IκBα and IKK α/β as well as their phosphorylated form. Representative western blots were shown (C). The relative densities of p-IKK α/β, p-IκBα, p-NF-κB p65 bands normalized to GAPDH were calculated (E, F and G).

Given that DR5 expression was shown to be regulated by the nuclear factor kappa-light-chain-enhancer of activated B cells (NF-κB) pathway[12], we next investigated the role of NF-κB pathway in regulating DR5 expression in the four glioblastoma cell lines. We used Bay 11–7082 to specifically inhibit NF-κB activation by blocking phosphorylation of I-κBα. Fig 2A and 2D indicated that DR5 expression was elevated in multiple glioblastoma cell lines. A low dose of lovastatin (2.5 μM) is sufficient to up-regulate DR5 expression in these cell lines. We next examined NF-κB activity in U87 cells treated with lovastatin using a dual-luciferase reporter assay. Fig 2B showed that 20 μM lovastatin significantly reduced NF-κB activity in U87 cells (Fig 2B).

Time-course experiments revealed that phosphorylated NF-κB p65, the active form of NF-κB p65, was decreased in U87 cells after treatment with lovastatin for 24 hours (Fig 2C and 2G). Moreover, the abundance of phosphorylated form of IKKα/β and I-κBα, which control the activation of NF-κB, were also down-regulated by lovastatin (Fig 2C, 2E and 2F). Taken together, our luciferase reporter assay and immunoblotting consistently indicated that lovastatin can inhibit NF-κB pathway and it suggested that lovastatin may promote DR5 expression via inhibition of NF-κB pathway.

### Lovastatin activates JNK pathway and inhibits MAPK/Erk pathway in glioblastoma cell lines

**Fig 3:**
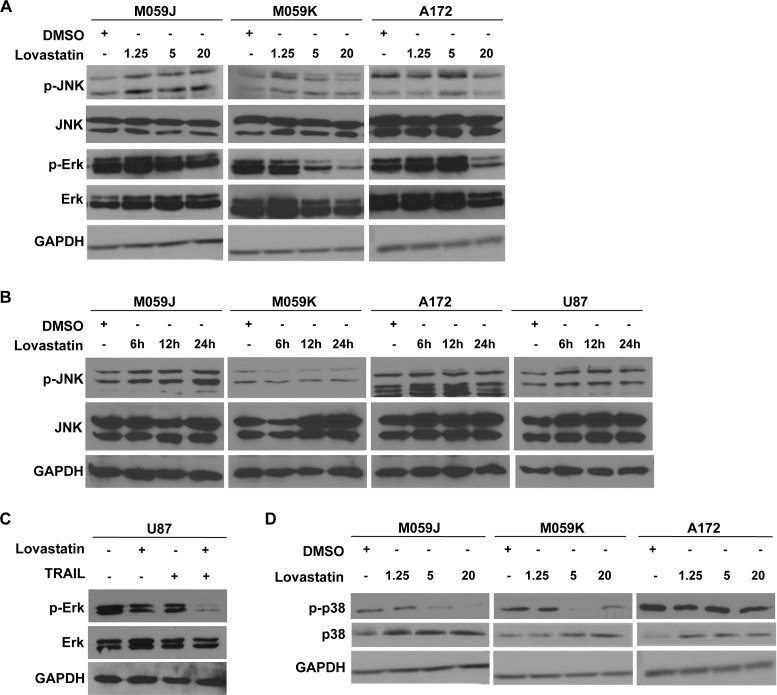
Lovastatin treatment in glioblastoma cell lines promotes JNK pathway and inhibits MAPK/Erk pathway. (A) The glioblastoma cell lines were treated with different concentrations of lovastatin. After treatment for 24 hours, the protein expression of JNK and Erk in the cell lines were detected by western blot. Representative western blots were shown. (B) The glioblastoma cell lines were treated with 20 μM of lovastatin for different treatment time. The expression of JNK was examined by western blot. (C) U87 cell line was treated with 20 μM of lovastatin or 100 ng/ml of TRAIL. After 24 hours treatment, the expression of Erk was detected. (D) Expression of p38 was detected in glioblastoma cell lines treated with different concentrations of lovastatin for 24 hours.

In addition to NF-κB, several signaling pathways such as protein kinase B/Akt and mitogen-activated protein kinase (MAPK) pathways are also implicated in TRAIL resistance[13]. The activation of MAPK signaling including c-Jun N-terminal kinase (JNK) and p38 MAPK pathways have been shown to associate with induction of apoptosis by stress agents[14–16]. We, therefore, investigated the effects of lovastatin treatment on activation of MAPK signaling. In the four glioblastoma cell lines treated with lovastatin for 24 hrs, JNK activation was enhanced in M059J, A172 and U87 cell lines (Fig 3A and 3B). We also treated the glioblastoma cell lines with lovastatin for different time, and found that treatment for 6 hours was able to enhance JNK activation in M059J and U87 cell lines (Fig 3B). After treatment with lovastatin, phosphorylation of Erk was significantly down-regulated in the four glioblastoma cell lines (Fig 3A and 3C). Considering the synergistic effect of lovastatin and TRAIL in inducing apoptotic cell death, we also examined Erk activation in U87 cells treated with lovastatin and TRAIL. Compared with single treatment, combined treatment with lovastatin and TRAIL can efficiently down-regulate Erk activation, indicating a synergistic effect of the two chemicals in inhibiting Erk activation (Fig 3C). However, activation of p38 was not detected after treatment with lovastatin (Fig 3D). These data suggested that activation of JNK as well as inhibition of Erk1/2 activation could underlie the effects of lovastatin treatment in the glioblastoma cell lines.

### Lovastatin inhibits tumor growth in mouse models carrying subcutaneous brain tumor

**Fig 4:**
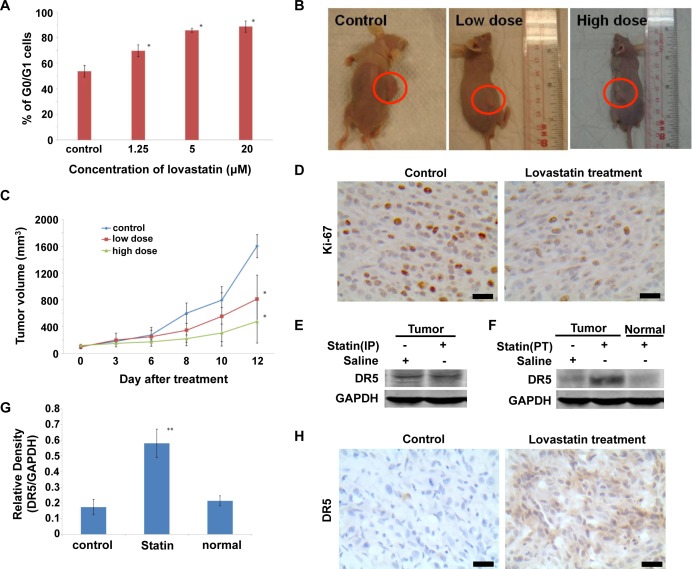
Anti-tumor effect of lovastatin on glioblastoma animal model and induction of DR5 expression by peri-tumoral administration of lovastatin in nude mice. (A) The U87 MG cells were treated with different concentrations of lovastatin for 48 hours, and then cell cycle progression was analyzed by PI staining. (B-E) The subcutaneously brain tumor nude mice were randomly divided into three groups with 10 mice in each group. The mice received intraperitoneal administration of none, low (5 mg/kg • day) or high (10 mg/kg • day) dose of lovastatin treatment for 12 days. (C) Tumor volumes in the subcutaneous brain tumor mouse model receiving different lovastatin treatments were shown (* *p* < 0.05). (D) After treatment of lovastatin, the nude mice were sacrificed and tumor specimens were collected. The expression of Ki-67 was determined by immunohistochemical staining. The brown crystals that could be seen in the cell nuclei were the positive signal of Ki-67. Scale bar = 40 μm. The DR5 expression was determined by western blot (E). Statin(IP), intraperitoneal administration of lovastatin. (F-H) The subcutaneously brain tumor nude mice were randomly divided into two groups, including control group and lovastatin group (10 mg/Kg • day). All animals received placebo or lovastatin daily by peri-tumoral injection for 12 days. After treatment, the tumor tissue was harvested and DR5 expression was measured using western blot (F-G) and immunohistochemical staining (H). The liver tissue from lovastatin treated mice served as control in western blot. Statin(PT), peri-tumoral injection of lovastatin. (G) The target bands were normalized to GAPDH and the index of densities was calculated. Scale bar = 20 μm in (H).

We have previously reported that lovastatin treatment promotes G0-G1 phase arrest in M059K, M059J, and A172 cell lines[7]. Here, we examined cell cycle progression in U87 cells treated with lovastatin. Fig 4A showed that lovastatin treatment for 48 hours also promotes G0/G1 phase arrest in a dose-dependent manner (Fig 4A), indicating lovastatin can broadly suppress cell cycle in multiple glioblastoma cell lines. These results prompted us to investigate the *in vivo* effects of lovastatin on tumor growth using subcutaneous brain tumor animal models.

We first established a U87 cell line stably expressing Luciferase-GFP fusion protein using a lentiviral transduction system (S2 Fig). GFP signals allow determining the transduction efficiency, and luciferase activities serve as an optical readout of gene expression or tumorgenesis *in vivo*. As shown in S2A Fig, after two weeks of selection by blasticidin, over 90% of U87 cells were transduced with lenti-GFP-Luc, exhibiting a significantly higher luciferase activity compared to the untransduced control (S2A and S2B Fig). The transduced and untransduced U87 cells exhibited similar growth rate (S2C Fig). The transduced U87 cells were then implanted subcutaneously in nude mice to form palpable tumors in two weeks and the tumor volume has reached about 100 mm^3^ by one month (data not shown), which is the threshold for treatment. Then the mice carrying U87 tumors were divided into three groups which received none, low (5 mg/kg) or high (10 mg/kg) dose of lovastatin respectively by intraperitoneal injection daily for 12 days. The tumor growth was monitored with electronic calipers every 2 days. The lovastatin-treated mice did not exhibit any differences in body weight compared to non-treated group (data not shown). As shown in Fig 4B and 4C, the tumor size was similar in all three experimental groups up to 6 days post treatment; however, starting from the second week onwards, the tumors in both low- and high-dose lovastatin-treated groups grew slowly compared to the control group. The group receiving the high dose of lovastatin (10 mg/kg) developed the smallest tumor (Fig 4B and 4C).

We further examined both macroscopic and microscopic appearance of the tumor specimens. All samples appeared smooth, solid and with new blood vessel formation (Data not shown). Microvascular proliferation, cellular variety, and nuclear atypia were also observed by hatmatoxylin and eosin staining in both control and lovastatin treatment groups (Data not shown). The expression of Ki-67, a specific proliferation marker, was markedly decreased in the high-dose lovastatin treated group (Fig 4D), indicating the reduced proliferation of tumor cells.

### Local peri-tumoral administration of lovastatin inhibits tumor growth and promotes DR5 expression in subcutaneous brain tumor

Given that *in vivo* lovastatin treatment is sufficient to inhibit tumor growth *per se* (Fig 4B and 4C), and promotes DR5 expression *in vitro* (Fig 1D), we next examined DR5 expression in subcutaneous brain tumor tissues collected from mice treated with lovastatin. Intraperitoneal injection of lovastatin did not alter DR5 expression compared to the control group (Fig 4E). However, DR5 expression was significantly increased in tumor tissues when lovastatin was given through local peri-tumoral administration (Fig 4F–4H), while the expression was not altered in the normal tissue such as liver (Fig 4F and 4G), indicating that local peri-tumoral administration of lovastatin selectively induced DR5 expression in tumor tissue but not in the normal tissue.

### Lovastatin enhances the efficacy of TRAIL on subcutaneous brain tumors in mice models

**Fig 5:**
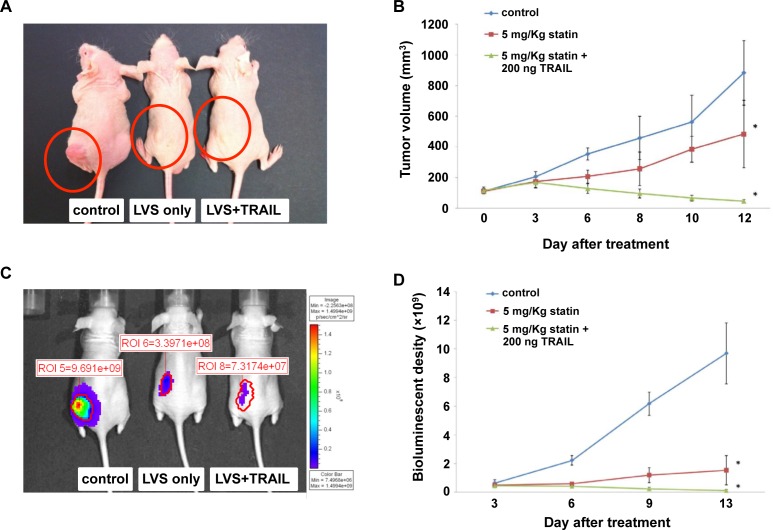
Lovastatin enhances the efficacy of TRAIL on subcutaneous brain tumor in mice models. The nude mice bearing subcutaneous brain tumor were randomly divided into control group, lovastatin group (5 mg/Kg • day lovastatin), and lovastatin plus TRAIL group (5 mg/Kg • day lovastatin plus 200 ng TRAIL). The nude mice received the lovastatin daily by peri-tumoral injection for 12 days. The nude mice of lovastatin plus TRAIL group were peri-tumorally administrated with TRAIL from day 4 to day 6, day 10 and day 12 post treatment. The subcutaneous tumor growth was observed by electronic calipers (A, B) and IVIS system (C, D).

In view of the role of lovastatin in sensitizing glioblastoma cell lines to TRAIL-induced apoptosis *in vitro* as well as in specifically inducing DR5 expression *in vivo*, we next tested whether lovastatin could enhance the efficacy of TRIAL on subcutaneous brain tumor in mice models. Volumes of the subcutaneous brain tumors were monitored by electronic calipers and IVIS system every 2–3 days for 12 days. As the result shown, in the mice treated with lovastatin alone or the combination of lovastatin and TRIAL, the tumor size was significantly smaller compared to the control group (Fig 5A and 5B). More notably, the combination treatment with lovastatin and TRAIL substantially reduced the tumor volume by 89% compared with the lovastatin treatment (Fig 5B). Furthermore, bioluminescence imaging by IVIS system consistently revealed that the tumor growth was markedly suppressed in the mice co-treated with lovastatin and TRAIL (Fig 5C and 5D). Taken together, these results suggested that the combined treatment with lovastatin and TRAIL can effectively inhibit tumor growth in mouse model of human glioblastoma.

## Discussion

TRAIL resistance is found in several types of human glioblastoma[17]. Given its selectivity to target cancer cells, TRAIL-based therapies still hold clinical potentials to cancer treatment in conjunction with other therapies or clinically proven drugs[18–21]. Lovastatin, a commonly used cholesterol-lowering agent for prevention of atherosclerotic cardiovascular diseases[22], has been reported to inhibit proliferation of breast cancer cell lines[23] and induce apoptosis of ovarian cancer cells synergistically with doxorubicin[24]. We have previously reported that lovastatin sensitized human glioblastoma cells to TRAIL-induced apoptosis and caused cell cycle arrest at G0/G1 phase[7]. In the present study, we further investigated the molecular basis underlying the effects of lovastatin in the sensitization of TRAIL-mediated apoptosis in the glioblastoma cells, and tested pre-clinically the efficacy of combined treatment of lovastatin and TRAIL on subcutaneous brain tumors in mice. We found that lovastatin was sufficient to induce TRAIL-mediated apoptosis in the four human glioblastoma cells that are resistant to TRAIL-based chemotherapy. We also found that DR5 expression was increased upon lovastatin treatment which provides a molecular basis by which lovastatin enhances TRAIL-mediated apoptosis. In the *in vivo* subcutaneous brain tumor mice models, we found that DR5 expression was also elevated in the tumor tissue collected from mice that had received lovastatin through local peri-tumoral administration while remained unchanged in the normal tissues, indicating the specific effects of lovastatin on tumor tissues. Furthermore, in the mouse models, lovastatin in conjunction with TRAIL led to strikingly diminished tumor volumes as well as halted tumor growth. These findings suggest the roles of lovastatin in enhancing efficacies of TRAIL-based therapy.

In meningioma cells, lovastatin acts as a potent inhibitor of proliferation by reducing activation of Erk signaling pathway[25]. In this study, it is also worth noting that lovastatin alone was sufficient to inhibit tumor growth *in vivo* as evidenced by markedly reduced tumor size and expression of specific proliferation marker Ki-67, suggesting that lovastatin acts by either inducing apoptosis or inhibiting cell proliferation. Although it was reported that lovastatin activates an apoptotic pathway dependent on protein synthesis[26], not all apoptotic processes require protein synthesis[27]. As *in vitro* single treatment with lovastatin did not affect cell viability, it was suggested that lovastatin inhibit proliferation. Indeed, western blot analysis confirmed that in glioblastoma cell lines treated with lovastatin, phosphoryaltion of Erk was markedly reduced. Importantly, combined use of lovastatin and TRAIL can synergistically inhibit Erk activation *in vitro*, and this is consistent with the *in vivo* data that combined treatment with lovastatin and TRAIL can effectively inhibit tumor growth in mouse model of human glioblastoma.

**Fig 6:**
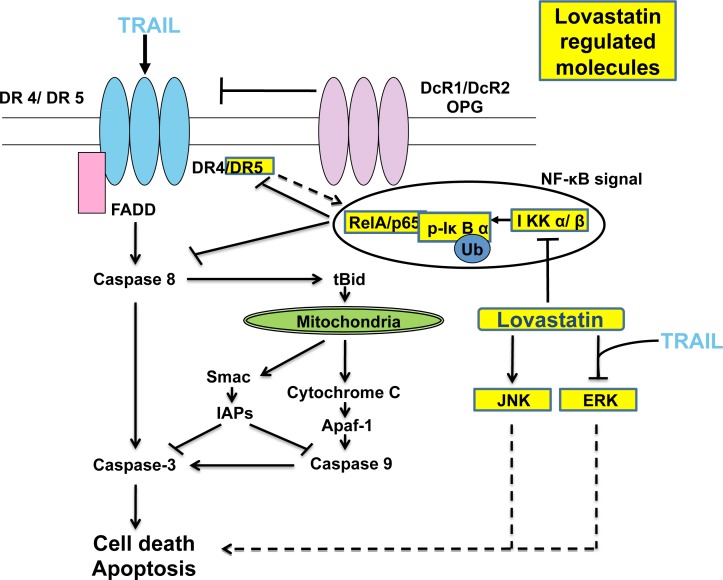
Schematic representation of the mechanism of TRAIL-induced apoptosis sensitized by lovastatin in human glioblastoma cells.

The activation of NF-κB pathway is implicated in the resistance to TRAIL-induced apoptosis[28, 29], and inhibition of NF-κB activation would unmask the TRAIL-mediated apoptotic signaling cascade. Recently it was also reported that bortezomib sensitizes malignant human glioma cells to TRAIL through inhibition of the NF-κB signaling pathway[30]. In addition, NF-κB was reported to regulate DR5 expression[12]. Our results demonstrated that lovastatin induced DR5 expression through inhibiting the activation of NF-κB. Apart from that, lovastatin also promoted cell apoptosis through down-regulation of ERK/MAPK expression and up-regulation of JNK expression. The proposed mechanism is graphically represented in Fig 6. However, detailed molecular interplay between NF-κB pathway and TRAIL-induced apoptosis signaling requires further investigation.

In a clinical trial testing the safety of high-dose lovastatin, 18 patients with glioma were treated with lovastatin at 20–30 mg/kg per day for one week. The result showed that high doses of lovastatin were well tolerated[31]. In our study, the nude mice were administrated with lovastatin at the dose of 5 or 10 mg/kg per day, which is less than the human tolerated dose, and is also within the maintenance dose range (10–80 mg per day) for hyperlipidemia in adult (lovastatin dosage guide with precautions-drugs.com). According to a previous study, lovastatin is lipophilic and can cross the blood-brain barrier[32]. Taken together, it is feasible to treat GBM patients with combination of lovastatin and TRAIL.

Several clinical trials have been conducted to evaluate the efficacy of stain administration in human cancer treatment. In one study, the patients with primary invasive breast cancer were treated with atorvastatin (80 mg/day). After two weeks’ treatment, ki67 was significantly down-regulated[33]. In our study, lovastatin treatment in glioblastoma animal model also reduced Ki67 level in tumor tissue. In a prospective study to identify potential biomarkers of simvastatin chemopreventive activity, the contralateral breast of high-risk women received simvastatin 40 mg daily for 24–48 weeks after completing all treatments. The results show that simvastatin significantly reduced estrone sulfate concentrations[34]. It was reported that the addition of lovastatin to thalidomide and dexamethasone improved overall survival and pregression-free survival in patients with relapsed or refractory myeloma[35]. In a prospective study, the patients affected by refractory colorectal tumors with mutant K-Ras received combined treatment with simvastatin, cetuximab and irinotecan, which exerted a positive outcome and increased their survival[36].

It has been shown that the combination of lovastatin with other anti-neoplastic drugs potentiates chemotherapy of tumors[30, 37]. Our *in vivo* data also demonstrated that pre-treatment with lovastatin significantly increased apoptosis induced by TRAIL in human glioblastoma multiforme mice models. Together with the *in vitro* data that lovastatin can enhance TRAIL-mediated apoptosis through up-regulation of DR5 as well as inhibition of NF-κB pathway, our results provided molecular basis and pre-clinical evidence that lovastatin potentiates efficacy of TRAIL-based therapy for the treatment of human glioblastoma.

## Supporting Information

S1 Fig Confirmation of transduced U87 MG cells expressing GFP and Luciferase. (A) The genes encoding GFP and Luciferase were integrated into the U87 MG genome via a lentivirus system. After selection for two weeks, the transduced U87 MG cells were observed under an optical microscope and a fluorescence microscope. (B) Bioluminescence assay of the transduced U87 MG cells and untransduced U87 MG. The assay was repeated at least three times using different passages of cells. U87-GFP-Luc, transduced U87 MG cells expressing GFP and luciferase. (C) Growth curves of transduced U87-GFP-Luc and untransduced U87 MG. (* p < 0.05).
(TIF)

S2 Fig IVIS imaging of the U87-GFP-Luc subcutaneous tumor model. Subcutaneous tumors were found in the dorsal area. The larger tumors showed higher luciferin intensity, indicating a positive correlation between tumor size and bioluminescent signal.
(TIF)

S1 File The original, uncropped and unadjusted blots generated for Fig 3. (DOC)

S2 File The original data for quantitative PCR shown in Fig 1B. (XLS)
