# Supplementary figures and images for: Inhibition of NF-κB Pathway and Modulation of MAPK Signaling Pathways in Glioblastoma and Implications for Lovastatin and Tumor Necrosis Factor-Related Apoptosis Inducing Ligand (TRAIL) Combination Therapy
Source: PLoS One. 2017 Jan 30;12(1):e0171157. doi: 10.1371/journal.pone.0171157 (PMC5279772; doi:10.1371/journal.pone.0171157)

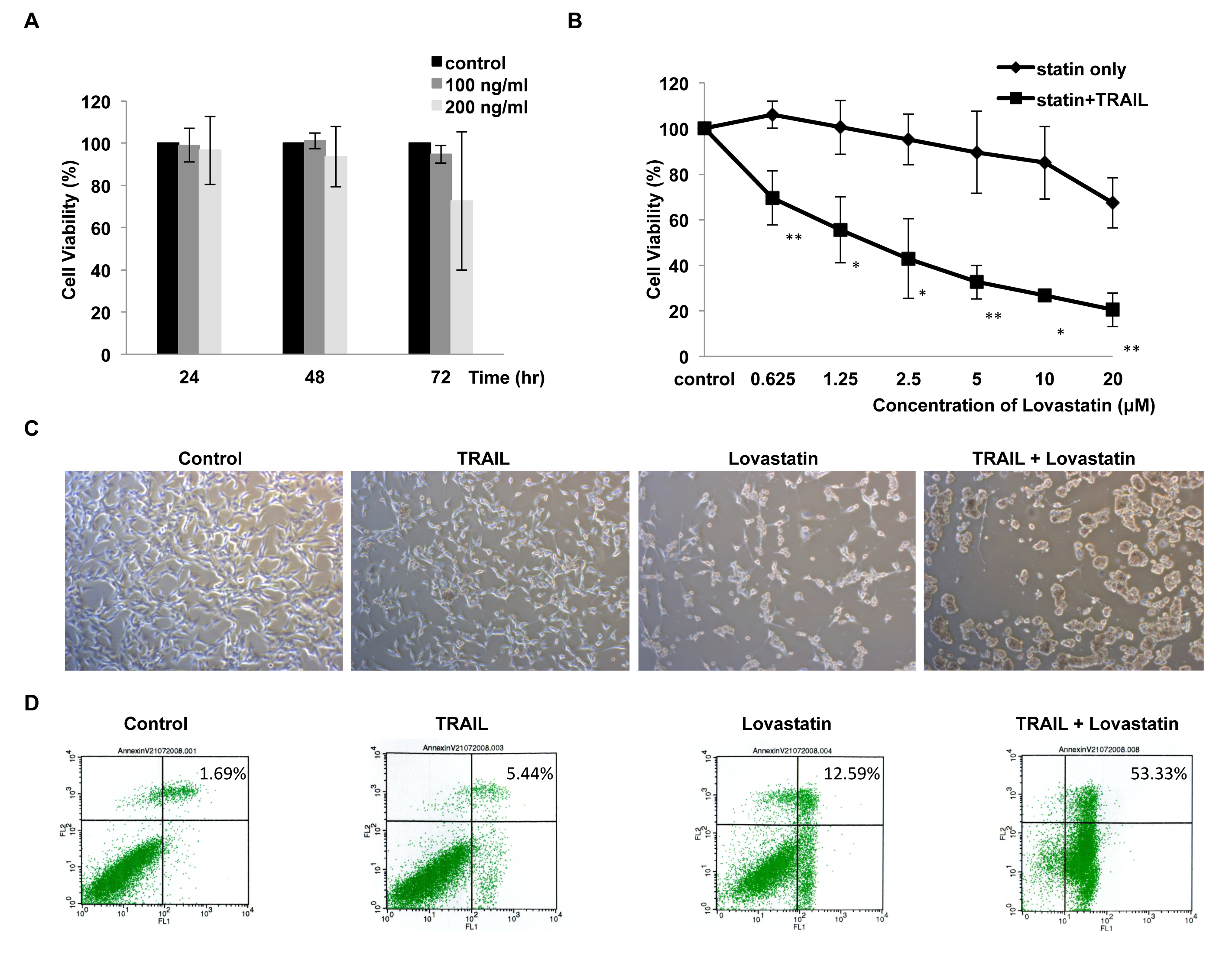

Supplement: S1 Fig — (A) The genes encoding GFP and Luciferase were integrated into the U87 MG genome via a lentivirus system. After selection for two weeks, the transduced U87 MG cells were observed under an optical microscope and a fluorescence microscope. (B) Bioluminescence assay of the transduced U87 MG cells and untransduced U87 MG. The assay was repeated at least three times using different passages of cells. U87-GFP-Luc, transduced U87 MG cells expressing GFP and luciferase. (C) Growth curves of transduced U87-GFP-Luc and untransduced U87 MG. (* p < 0.05). (TIF) [file pone.0171157.s001.tif]

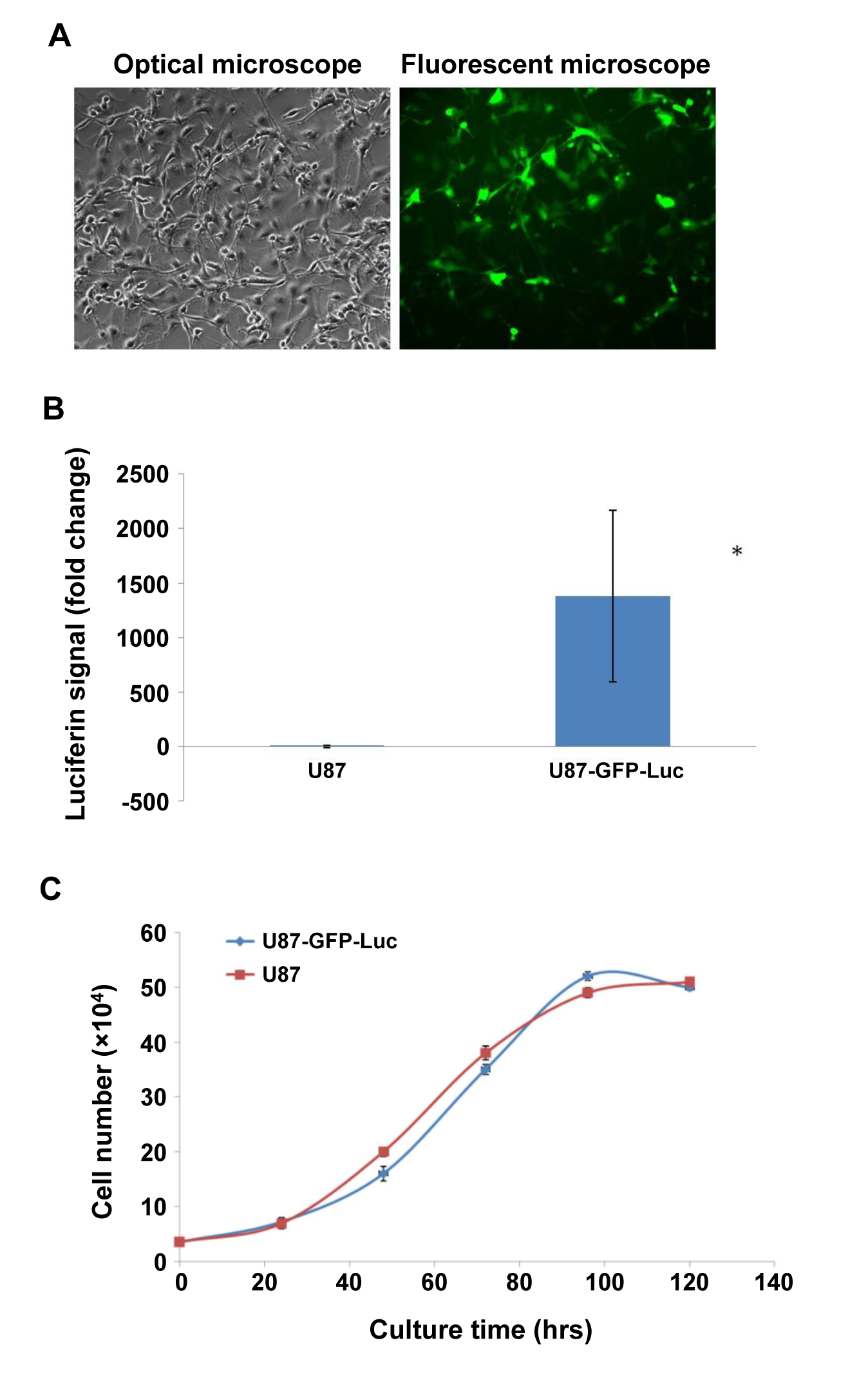

Supplement: S2 Fig — Subcutaneous tumors were found in the dorsal area. The larger tumors showed higher luciferin intensity, indicating a positive correlation between tumor size and bioluminescent signal. (TIF) [file pone.0171157.s002.tif]

Fig3A-total JNK


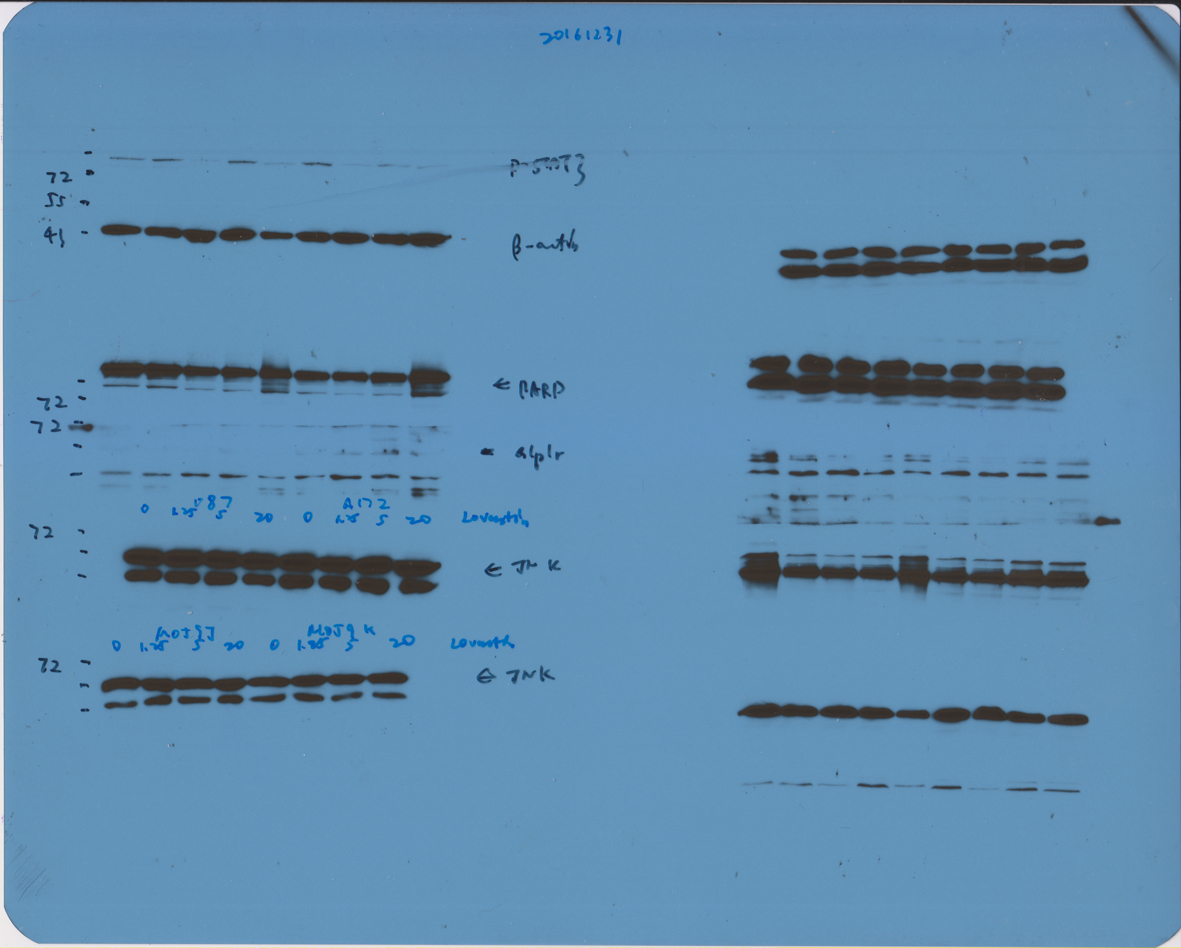


Fig3B-pJNK


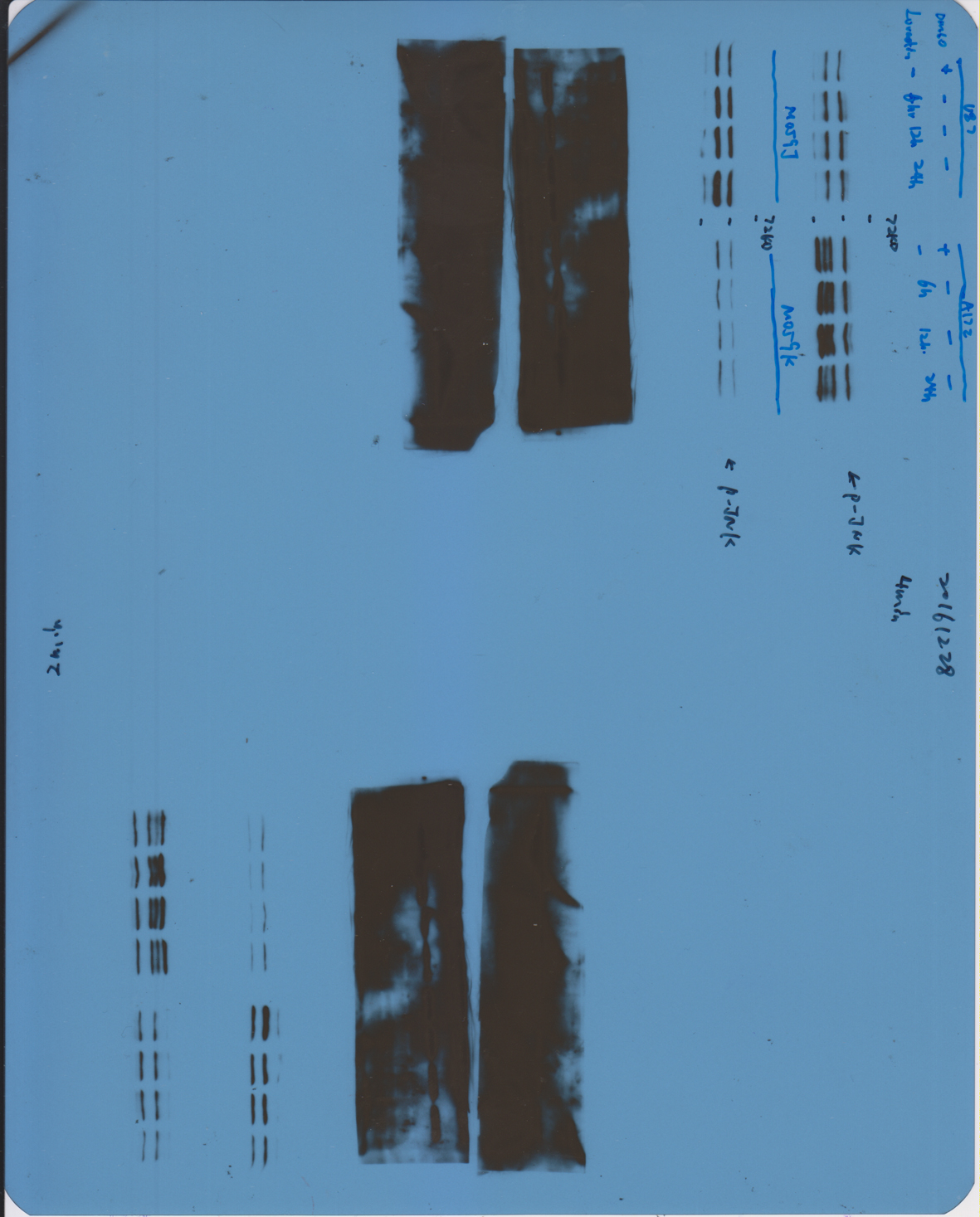


Fig3B-total JNK


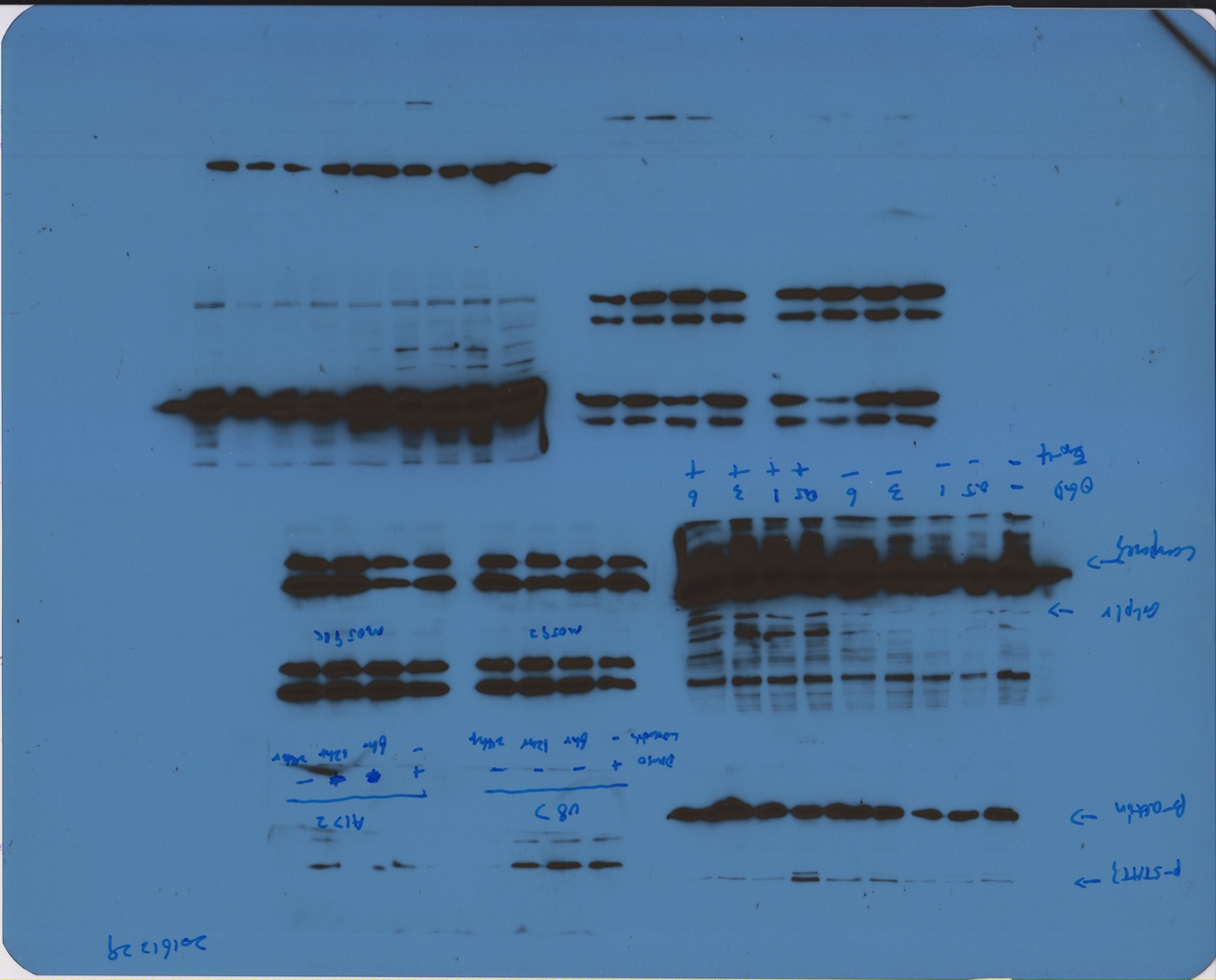


Fig3B-GAPDH


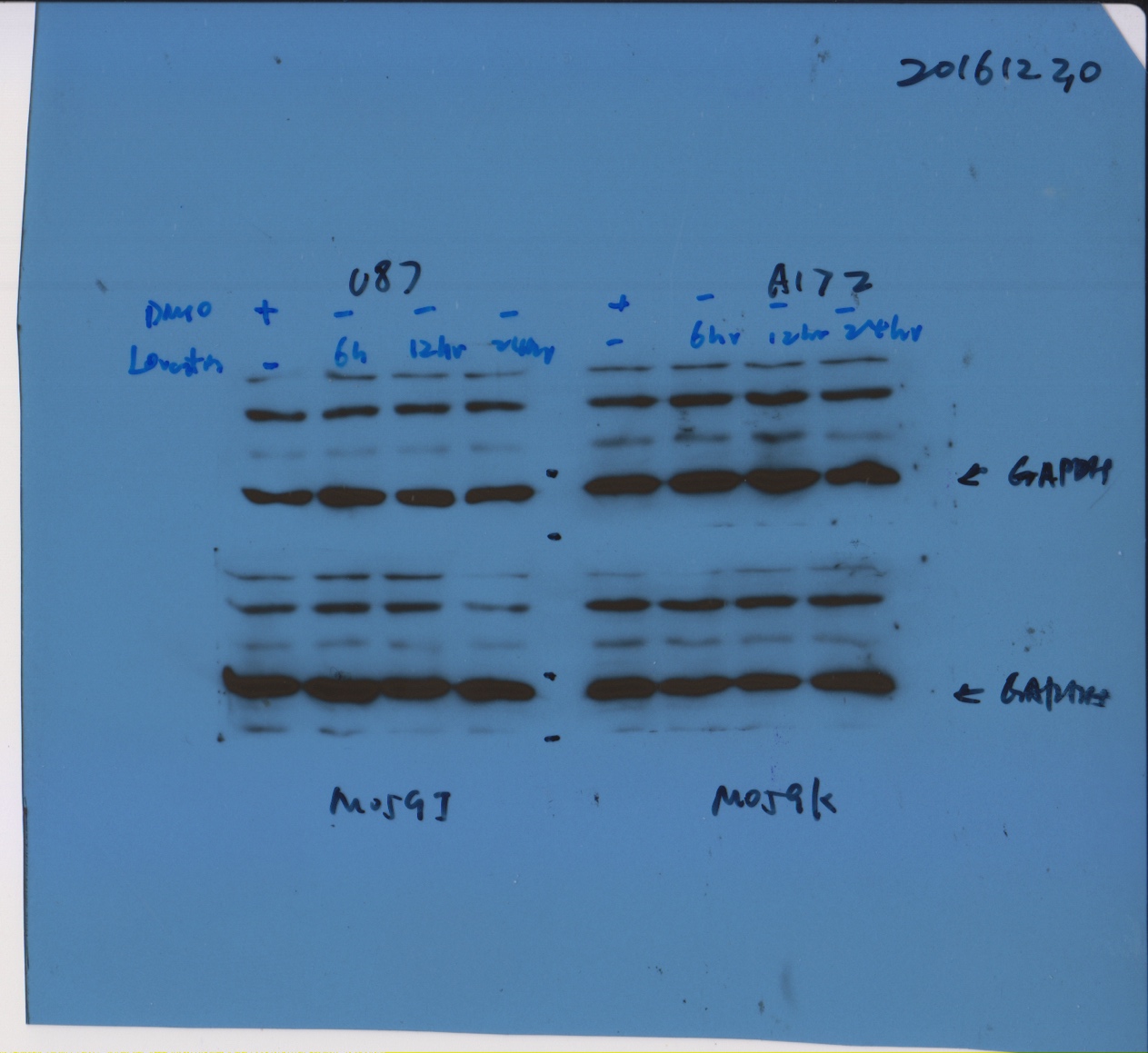


Fig3C


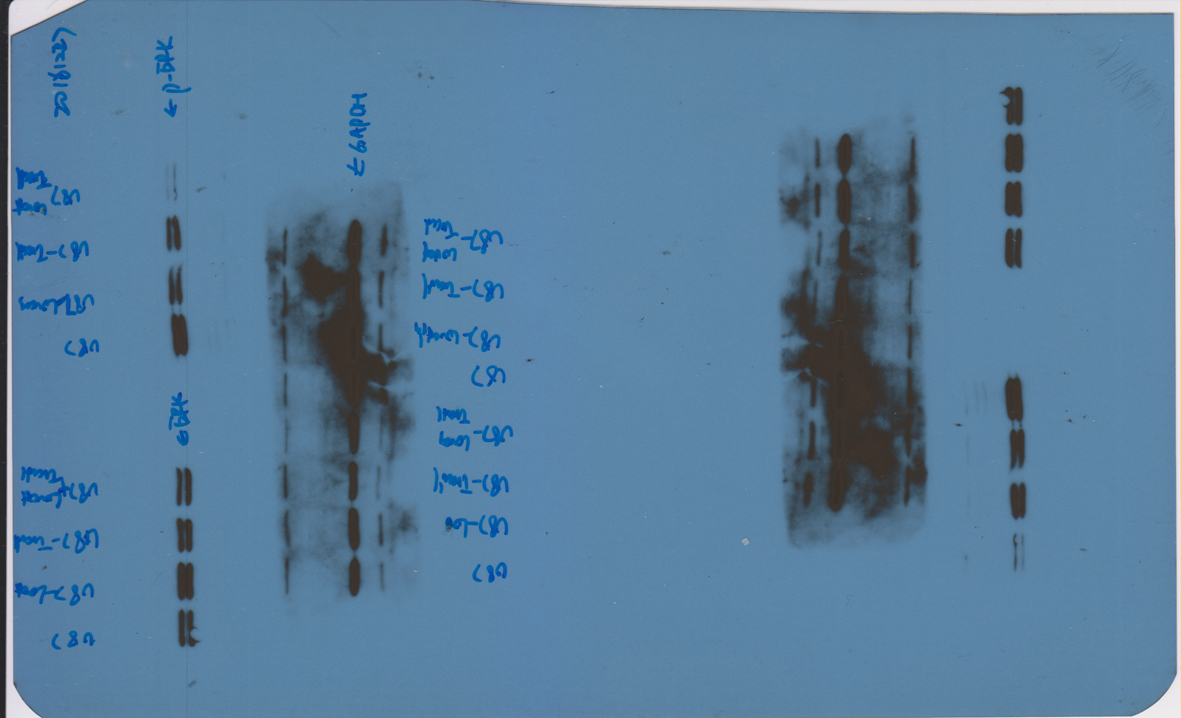


Fig3D-p-p38


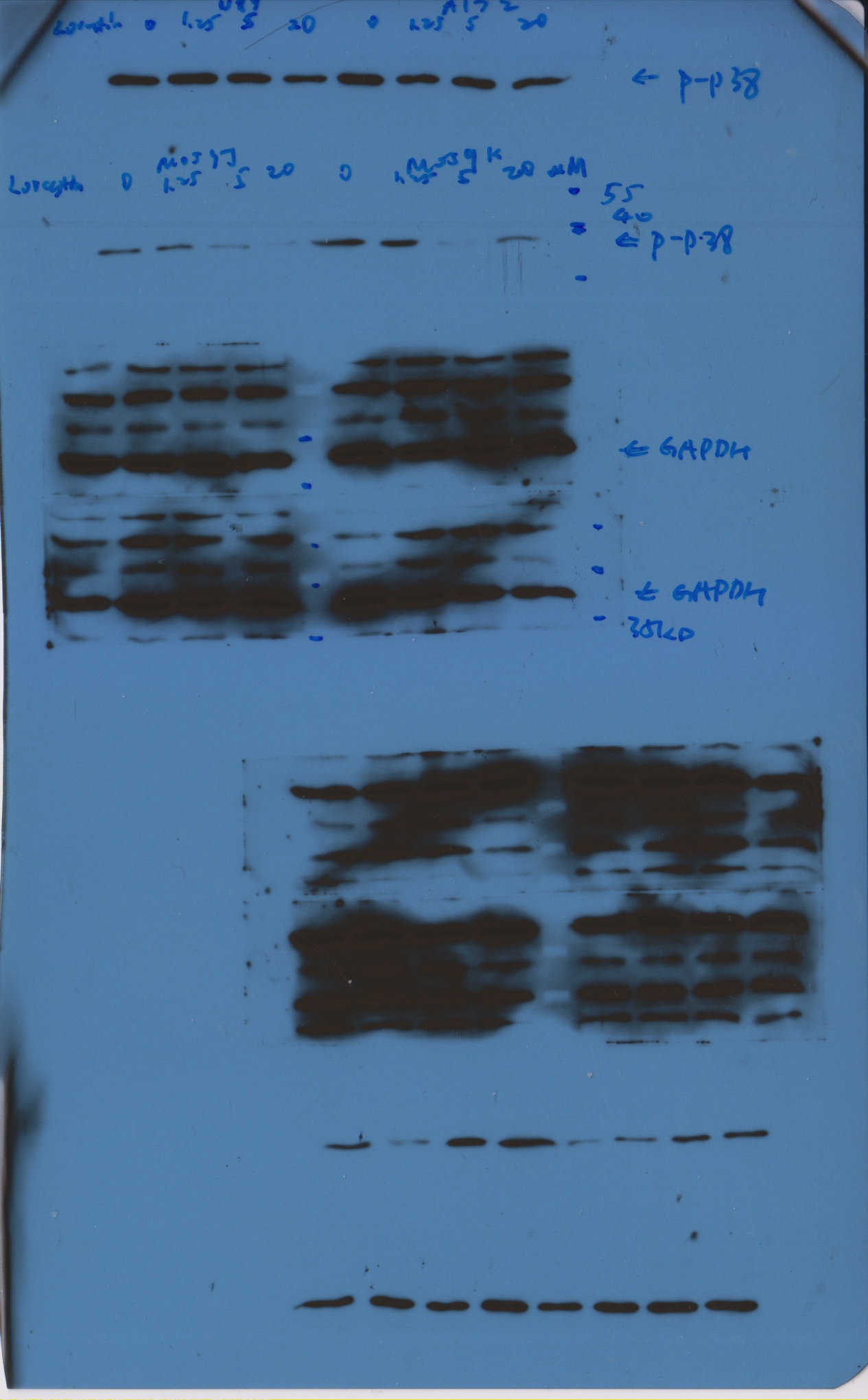

Supplement: S1 File — (DOC) [file pone.0171157.s003.doc]
